# Serum chymase levels correlate with severe dengue warning signs and clinical fluid accumulation in hospitalized pediatric patients

**DOI:** 10.1038/s41598-020-68844-z

**Published:** 2020-07-16

**Authors:** Abhay P. S. Rathore, Manouri Senanayake, Arjuna Salinda Athapathu, Sunethra Gunasena, Irantha Karunaratna, Wei Yee Leong, Ting Lim, Chinmay Kumar Mantri, Annelies Wilder-Smith, Ashley L. St. John

**Affiliations:** 1 Program in Emerging Infectious Diseases, Duke-National University of Singapore Medical School, 8 College Rd., Level 9, Singapore, Singapore; 2 Present Address: Department of Pathology, Duke University Medical Center, Durham, NC USA; 3 Department of Paediatrics, Faculty of Medicine, University of Colombo, Colombo, Sri Lanka; 4 Lady Ridgeway Children’s Hospital, Colombo, Sri Lanka; 5 Department of Virology, Medical Research Institute (MRI), Colombo, Sri Lanka; 6 Lee Kong Chian School of Medicine, Nanyang Technological University, Singapore, Singapore; 7 Department of Disease Control, London School of Hygiene and Tropical Medicine, London, UK; 8 Heidelberg Institute of Global Health, University of Heidelberg, Heidelberg, Germany; 9 Department of Microbiology and Immunology, Yong Loo Lin School of Medicine, National University of Singapore, Singapore, Singapore; 10 SingHealth Duke-NUS Global Health Institute, Singapore, Singapore

**Keywords:** Viral infection, Biomarkers, Predictive markers, Prognostic markers, Infection, Pathogens, Dengue virus

## Abstract

Dengue induces a spectrum of severity in humans from the milder dengue fever to severe disease, or dengue hemorrhagic fever (DHF). Chymase is a candidate biomarker that may aid dengue prognosis. This prospective study aimed to identify whether warning signs of severe dengue, including hypovolemia and fluid accumulation, were associated with elevated chymase. Serum chymase levels were quantified prospectively and longitudinally in hospitalized pediatric dengue patients in Sri Lanka. Warning signs were determined based on daily clinical assessments, laboratory tests and ultrasound findings. Chymase was significantly elevated during the acute phase of disease in DHF or Severe dengue, defined by either the 1997 or 2009 WHO diagnosis guidelines, and persisted longer in the most severe patients. Chymase levels were higher in patients with narrow pulse pressure and clinical warning signs such as severe leakage, fluid accumulation, pleural effusion, gall-bladder wall thickening and rapid haematocrit rise concurrent with thrombocytopenia. No association between chymase and liver enlargement was observed. This study confirms that serum chymase levels are associated with DHF/Severe dengue disease in hospitalized pediatric patients. Chymase levels correlate with warning signs of vascular dysfunction highlighting the possible functional role of chymase in vascular leakage during dengue.

## Introduction

Dengue virus (DENV) is one of the most important human pathogens and infects between 50 and 390 million people each year^1^. DENV is now endemic in over 100 countries worldwide and one estimate suggests that ~ 3.9 billion people are at risk for contracting the disease^2,3^. Increasingly, international travelers are also affected^4–7^. In Sri Lanka (the site for this study), a major outbreak of dengue occurred in 2017, resulting in ~ 185,000 clinical cases^8^. The burden of dengue is also high in other Southeast Asian countries including Malaysia, Thailand, Vietnam and the Philippines where there were nearly 1,400 DENV-related deaths reported in 2017 alone^9^. DENV is spread to humans by mosquito bites, primarily by *Aedes* aegypti, and causes a febrile illness known as dengue fever (DF). In some cases, patients can develop a severe and life-threatening form of dengue disease known as dengue hemorrhagic fever (DHF) and dengue shock syndrome (DSS)^10,11^. In 2009, the WHO published a re-classification of the dengue case classification as “dengue with or without warning signs” and “Severe dengue”^12^. Most DHF cases and all DSS cases are reclassified as Severe dengue in this new system.

DENV has four distinct serotypes, (DENV 1-4) and immunity to each is long-lasting and provides homotypic protection^13,14^. However, in DENV endemic regions, a patient can experience multiple DENV infections in a lifetime. A typical DENV infection is characterized by an incubation period of 4–7 days before fever onset. DF is an acute febrile illness that consists of fever, headache, myalgia, retro-orbital pain, nausea and rash. Leukopenia and mild thrombocytopenia are frequently observed in DF patients^15,16^. Hemorrhagic manifestations such as petechiae, purpura, gingival bleeding and gastrointestinal bleeding are also observed in some cases^17,18^. The febrile period lasts for 5–7 days before symptoms resolve. At the point when symptoms of febrile illness are resolving, some patients may experience severe and life threatening complications such as DHF and DSS^12,19^. DHF is characterized by high fever, thrombocytopenia and major bleeding (measured by a rise in hematocrit levels). An important pathology that differentiates DF from DHF is plasma leakage, where an increase in capillary permeability allows the transudate to collect at the pleural and abdominal cavities. Gall bladder wall thickening and ascites are also observed in DHF patients^20,21^. Without intervention, DHF can lead to circulatory failure, resulting in narrow pulse pressure and shock. The prognosis for fatal outcomes in DHF at the stage where shock is observed increases from 0.2% to 44%^10,22^. For DHF diagnosis a patient must meet 4 criteria: fever, hemorrhagic manifestations, thrombocytopenia (platelet count, < 100,000 platelets/mm^3^) and evidence of plasma leakage^11^. Warning signs defined in the 2009 WHO criteria that are associated with DENV infection include abdominal pain, persistent vomiting, fluid accumulation, mucosal bleeding, lethargy, liver enlargement and the observation of increasing hematocrit combined with rapidly decreasing platelets. It is recommended that patients admitted with warning signs should be strictly observed and that medical intervention be promptly instituted. However, Severe dengue is diagnosed when a patient displays any of the following clinical signs: severe plasma leakage leading to shock or respiratory distress, severe hemorrhaging, or organ failure. Severe dengue could potentially be fatal and, therefore, urgent medical care is needed^12^. Warning signs appear late in DENV disease, usually around day 5 post-fever onset, making it extremely difficult to predict DHF/DSS or Severe dengue in the early acute/febrile phase of disease. Other factors, such as high viremia and high levels of viral secretory protein NS1 have been suggested to be associated with DHF/DSS, but with conflicting reports^23–32^.

We previously reported that elevated serum chymase, a mast cell (MC)-specific protease, is highly predictive of DHF in both adult and pediatric dengue patients in Sri Lanka and Indonesia when measured within 3–6 days of fever onset (acute phase of disease)^33,34^. We also observed that chymase was not elevated in non-DENV febrile patients, most of which had respiratory infections, compared to healthy controls^35^. Chymase is a serine protease and angiotensin-converting enzyme that can induce vascular permeability^36^. It is released by MCs when they are triggered to degranulate, which can be induced in response to DENV binding to the cells through an unknown receptor^35^ or in response to virus/antibody immune complexes through activating receptors such as FcγRIII or FcεRI^37^. In this prospective study, we examined the association of chymase with signs of vascular leakage in hospitalized pediatric patients. Here, we tested whether elevated chymase is observed in pediatric patients meeting the criteria of having DHF or Severe dengue, compared to the patients with DF or mild dengue. Furthermore, we hypothesized that chymase levels would be elevated in patients experiencing signs of vascular leakage and fluid accumulation, such as pleural effusion and gall bladder wall thickening.

## Methods

### Recruitment of dengue patients and sample collection

Pediatric dengue patients 5–12 years of age were prospectively recruited upon parental consent at the Lady Ridgeway Hospital, Colombo, Sri Lanka. Criteria for recruitment included onset of fever (≥ 38 °C for less than 72 h), hospital admission, and confirmation of DENV infection by NS1 antigen test (using the SD Dengue NS1 Ag + Ab Combo Dengue Duo, Standard diagnostics inc, South Korea) at the time of recruitment. Patients with thalassemia, hemophilia, heart disease, cancer, any bleeding disorder, CKD/any renal disease, and immuno-suppressed subjects were excluded. As we found that recruitment was difficult due to the strict entry criteria of 72 h (3 days after onset of fever), we relaxed the entry criteria to 96 h (4 days after onset of fever) and expanded the ages recruited to include children < 5 years. All NS1 positive DENV infections were confirmed by real time RT-PCR. A total of 84 cases were selected for laboratory testing for chymase, based on DENV confirmation and sufficient serum provided to complete the required molecular tests, with 45 DF cases and 39 DHF cases as a final diagnosis. Three blood samples were taken (1–3 days post fever onset, 4–5 days post fever onset and 6–7 days post fever onset) in BD Vacutainer SST tubes. Serum was isolated from these 3 sequential samples for DENV confirmation, quantification of viral RNA and chymase ELISA. Serum was isolated within 4 h of blood collection and stored at − 80 °C. Criteria for assessing the clinical diagnosis as DF or DHF (according to the 1997 WHO criteria) or as dengue with or without warning signs or Severe dengue (according to the 2009 WHO criteria) were recorded in the case report forms and were reviewed by multiple study team members who were not involved in the blinded laboratory analysis. Study questionnaires were filled by study team members based on patient interviews. Ultrasounds were performed as per the hospital standard protocol for dengue management, daily, from the first full day of hospitalization. The laboratory and clinical records were transcribed from the case notes of the patients into the case report forms by a trained clinician.

Research was designed to be compliant with the Declaration of Helsinki. Ethical approvals were obtained from the Institutional Review Boards of Lee Kong Chian School of Medicine, Nanyang Technological University (NTU), Singapore, National University of Singapore and the Ethics Review Committee of Faculty of Medicine, University of Colombo, Sri Lanka. Written informed consent was given by a parent or guardian of the patient.

### Serum chymase quantification

Human mast cell chymase I (CMA-I) kit (BlueGene Biotech, catalogue number E01M0368) was used to measure chymase levels in the patients’ serum by following the manufacturer’s instructions. Samples were blinded for chymase ELISA and viral quantification.

### Confirmation and quantification of DENV infection

Viral RNA was isolated from 140 μl of serum using the QIAamp Viral RNA Mini Kit (catalogue number: 52906) according to manufacturer’s instructions. PCR primers used to quantitate DENV1-4 from human sera were reference reagents obtained from the US Centers for Disease Control (CDC) and Prevention (catalog number: KK0129) and were used following the manufacturer’s protocol, according to the published method^38^. Invitrogen SuperScript III Platinum One-Step qRT-PCR kit (catalog number: 11732088) was also used in combination with the CDC reference reagents as instructed by the CDC method. Standard curves for viral quantification were generated using plasmids containing the DENV sequence of interest for DENV1-4 and viral titers are represented as genome copies per ml. To determine primary versus secondary DENV infections, DENV-specific IgM and IgG were measured in the acute serum samples. For this, the Panbio Dengue IgG Capture ELISA (Catalog number: 01PE10) and Panbio Dengue IgM Capture ELISA (Catalog number: 01PE20), both from PanBio Diagnostics, were used. Primary cases were identified as IgM^+^IgG^−^ and secondary cases were identified as IgM^+^IgG^+^^39^.

### Data analysis

Demographic statistics were generated using SPSS software (IBM). Prism software was used to compare mean chymase levels by 1- or 2-way ANOVAs. SPSS was also used for post-hoc power analysis. Results were considered significant at a *p* value < 0.05. After the selection of samples based on the inclusion criteria and dengue confirmation, no data were excluded. Unblinding and data analysis were performed only after all laboratory analyses were completed.

## Results

### Characteristics of patient cohort and DENV infections

**Figure 1:**
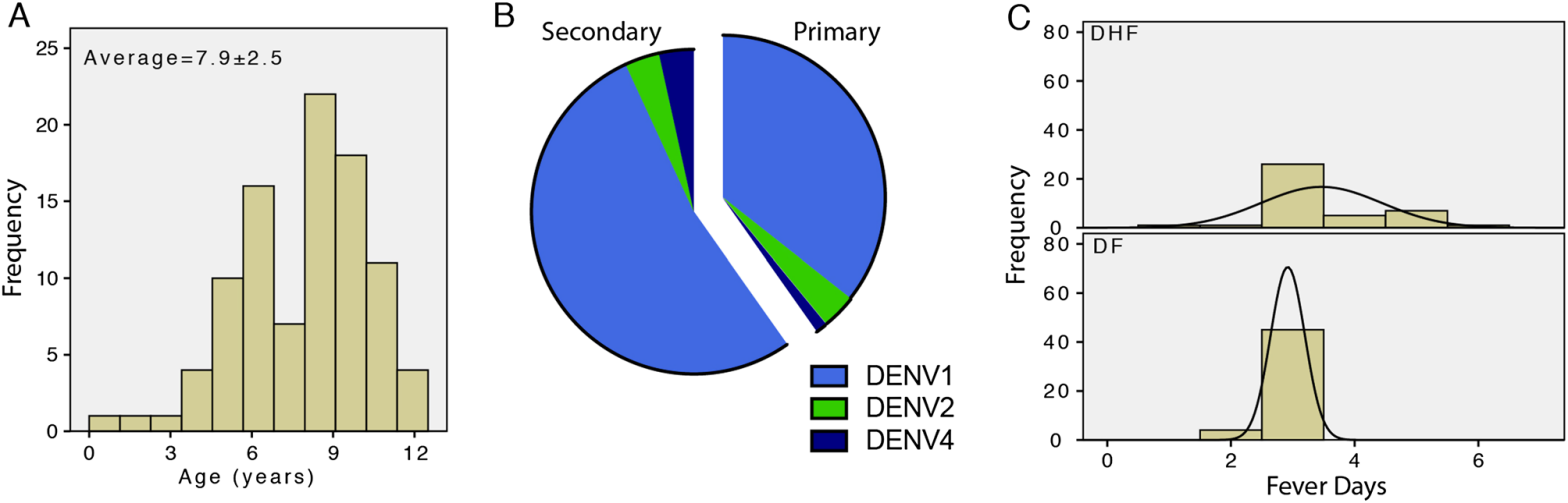
Characteristics of DENV infection and recruitment of patients. (**a**) Age distribution of subjects enrolled in the study and confirmed DENV-positive. (**b**) Chart depicts the proportions of primary and secondary DENV cases and the serotypes of DENV that were confirmed by PCR. (**c**) Histograms depict the numbers of patients recruited (frequency) for each day post-fever onset. The x-axis represents the number of days between fever onset and patient enrollment for DHF (n = 39) and DF (n = 45) patients. Black lines indicate the fitted curves for each histogram.

A total of 91 dengue NS1 positive febrile children recruited into the study were confirmed as DENV-positive in subsequent laboratory tests (DENV PCR positive) and 84 had provided sufficient serum for completing the test for chymase detection. Study subjects were under 12 years of age (Fig. 1a) and were 61% male, 39% female. A majority of DENV cases were secondary DENV infections, with 62% secondary infections and 38% primary DENV infections (Fig. 1b), determined by serology. Consistent with the emergence of DENV1 as the dominant epidemic serotype in the region since 2009^40^, the predominant serotype responsible for DENV infection in this cohort was DENV1, followed by DENV2 and DENV4 (Fig. 1b). Final diagnosis was assessed at the completion of the study based on clinical and laboratory assessments and none of the patients included met the case definition for DHF at the time of enrollment. Overall, the rates of DHF in this hospitalized cohort were high, with 46% (n = 39 DHF of n = 84 total) of study subjects receiving the final diagnosis of DHF, likely due to the fact that mild disease that did not require hospitalization was not represented in the cohort. Importantly, we noted that some DHF patients were recruited later in the course of disease compared to DF patients (Fig. 1c). We stratified all analyses based on day post fever onset to accurately reflect the kinetics of viremia and serum biomarker levels over time. Three blood samples were taken for each patient, with 93% retention for the second sample and 63% retention for the third sample. Drop-out was primarily due to recovery from DENV disease and withdrawal from the study after discharge from the hospital. There were no DENV-associated deaths in this cohort.

### Elevated early chymase correlates with severe dengue and DHF

**Figure 2:**
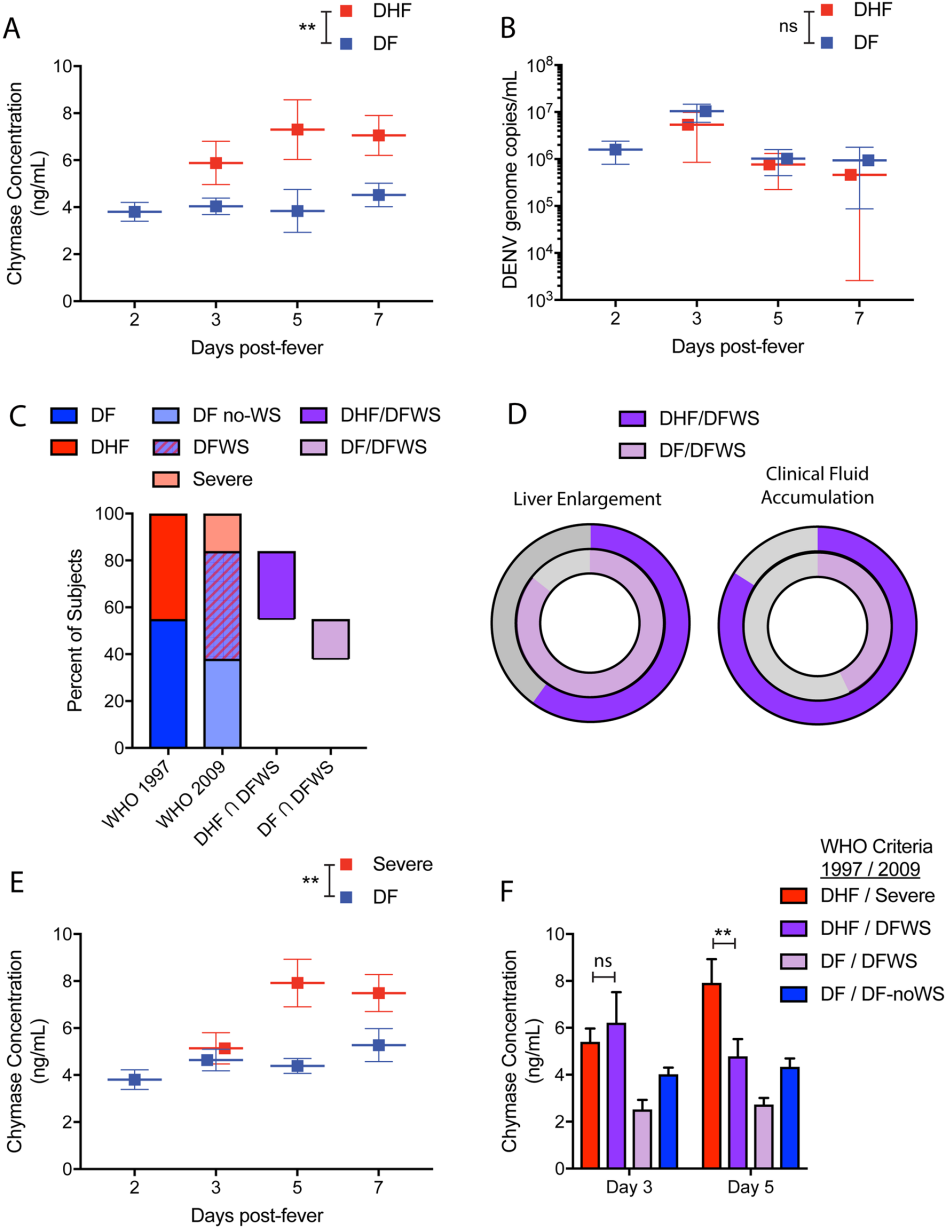
Serum chymase levels are prognostic of DHF and Severe dengue and peak later in Severe dengue patients. (**a**) Comparisons of serum chymase concentrations between DHF and DF patients (defined according to the WHO 1997 criteria), stratified by day post-fever onset. Chymase levels are elevated in DHF patients compared to DF patients, *p* = 0.0076, analyzed by 2-way ANOVA for days 3–7. (**b**) Serum virus titers (genome copies per mL of serum), determined by RT-PCR did not differ between DHF and DF patients (defined by WHO 1997 criteria). (**c**) Comparison of percentages of patients meeting the WHO 1997 diagnosis criteria for DHF versus DF, WHO 2009 diagnosis criteria for Severe dengue versus DF (with or without clinical warning signs). Those DHF patients that did not meet the WHO 2009 criteria for Severe dengue met the criteria for DFWS (n = 15). Some patients that met the 1997 criteria for DF met the criteria for DFWS. (n = 8) (**d**) Overlapping pie charts compare the proportions of DHF/DFWS and DF/DFWS patients displaying the warning signs of liver enlargement and clinical fluid accumulation. (**e**) Comparisons of serum chymase concentrations between Severe dengue and DF patients (defined according to the WHO 2009 criteria), stratified by day post-fever onset. (**f**) Concentrations of serum chymase amongst groups divided based on severity according to both 1997 and 2009 criteria for Severe dengue disease. Chymase levels in the serum do not distinguish between DHF/Severe and DHF/DFWS groups on day 3 post-fever onset, but chymase levels of DHF/Severe patients are significantly elevated over DHF/DF-WS patients on day 5 post fever onset. Comparisons amongst groups were performed by ANOVA with Sidak’s multiple comparison test. Error bars represent the SEM and ** indicates *p* < 0.01.

We measured chymase in the prospectively obtained serum samples and analyzed stratified based on the day of fever onset, which was determined by questionnaire. A significant increase in the levels of chymase was observed at day 3 post fever onset for the patients whose final diagnosis was DHF compared to a final diagnosis of DF (Fig. 2a). Chymase was elevated in the serum of both DF and DHF patients compared to healthy controls (Fig. S1). Viral titers in the serum did not correlate with DHF and were not significantly different between DHF and DF patients at any time points tested post fever onset (Fig. 2b). Viremia was reduced on days 5 and 7 compared to day 3 and likely resolving (Fig. 2b).

Since 2009, the WHO has recommended classifying dengue disease as Severe dengue, DF with warning signs (DFWS), or DF without warning signs (DF no-WS)^12^. Therefore, although this scheme is not routinely employed for clinical assessment in Sri Lanka, we also reclassified our patient cohort following the 2009 guidelines. This resulted in 16% of patients in our hospitalized cohort being classified as Severe dengue (Fig. 2c). We also found that 29% of total study subjects who experienced DHF, according the 1997 criteria, fell in to the DFWS category according to the 2009 criteria (Fig. 2c), while another 15% of patients with a DF diagnosis by the 1997 criteria also had at least one warning sign (Fig. 2c). This group of patients with DF/DFWS according to the 1997/2009 criteria, respectively, had a higher incidence of liver enlargement and a lower incidence of clinical fluid accumulation than the patients falling into the categories of DHF/DFWS (by the 1997 and 2009 criteria, respectively) (Fig. 2d). Consistent with the results of analysis using the 1997 classification (Fig. 2a), data reanalysis using 2009 criteria showed that chymase levels were significantly higher in the sera of Severe dengue patients compared to DF patients (Fig. 2e). Since reclassification of patients from the 1997 to 2009 WHO schemes effectively defines the less severe DHF patients as DFWS, we compared chymase levels between DHF patients in those two groups (DHF/DFWS vs. DHF/Severe by the 1997/2009 classifications). Patients with Severe dengue were not distinguishable from patients in the DHF/DFWS category at the early time point of 3 days post-fever onset (Fig. 2f). Yet, by day 5 post-fever onset, patients with Severe dengue (DHF/Severe) had significantly higher levels of serum chymase compared to patients meeting the criteria for DHF/DFWS diagnosis (Fig. 2f). This suggests that the processes that underlie exacerbated severity late in the time course of DENV disease are correlated with high chymase levels. Importantly, for both comparisons of DHF versus DF and Severe dengue versus DF (with and without warning signs), when chymase levels were analyzed relative to the day of enrollment rather than self-reported fever onset, significantly elevated chymase was still observed to correlate with severity (Fig. S2). We also noted that chymase levels were higher for DF patients in this study, compared to a previous Sri Lankan cohort we examined where non-hospitalized patients were also included^33^. However, average chymase levels for the DF patients (which were all hospitalized prior to recruitment) in this study were similar to the levels observed in pediatric patients who displayed signs of bleeding in that prior study (Fig. S3). Also similar to previous observations^35^, we did not observe augmented chymase levels in secondary DENV infection of DF patients, but did observe higher chymase levels in secondary DENV patients that were subsequently diagnosed with DHF (Fig. S4). These data indicate that Severe disease, defined by multiple objective clinical criteria, is strongly associated with elevated serum chymase. Longitudinal assessment of serum chymase concentrations indicates that it is elevated throughout the acute disease phase and persists at high levels longer in the serum of the most severe patients (Fig. 2).

### Clinical parameters associated with DHF and their correlation with chymase

**Figure 3:**
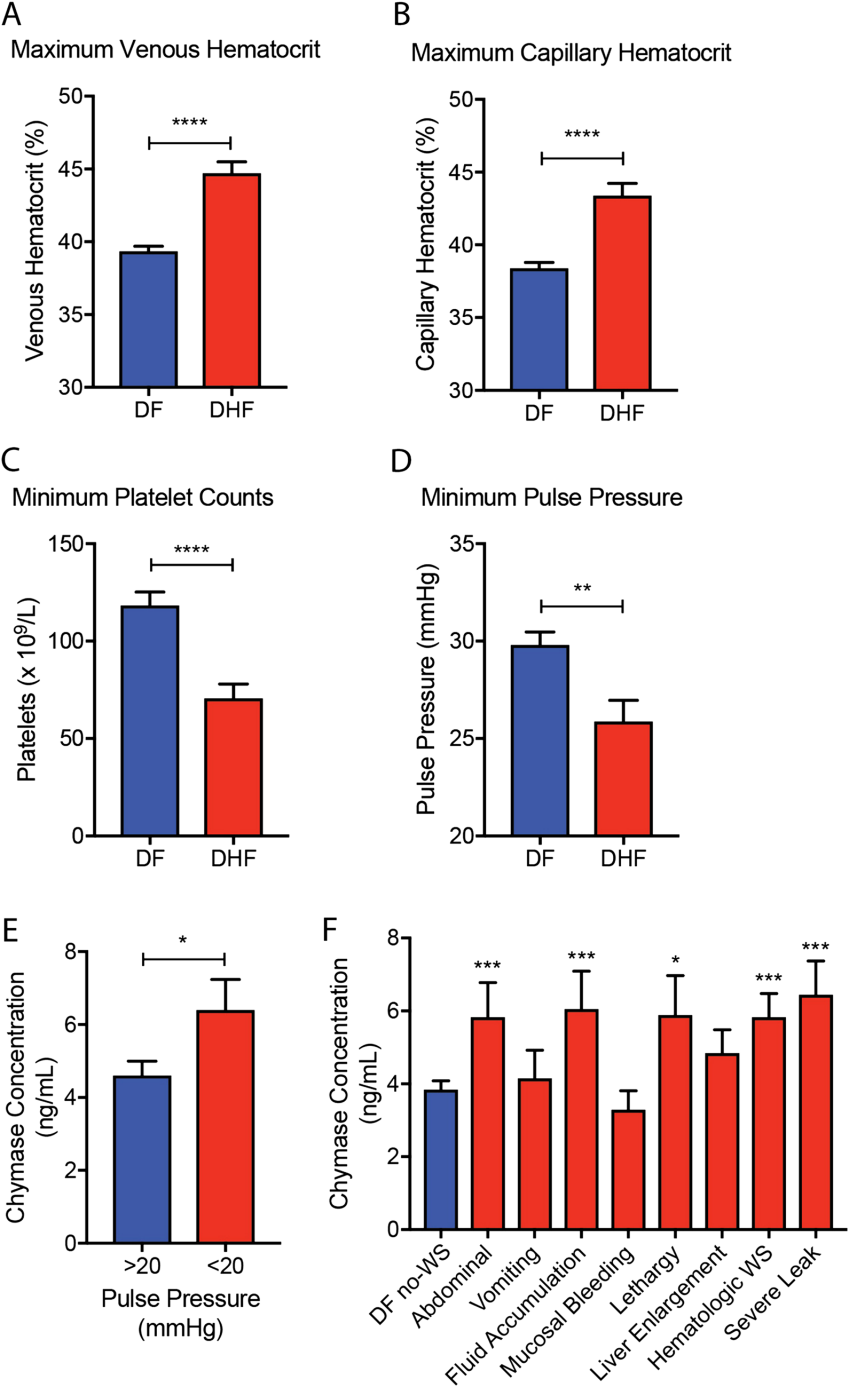
Clinical parameters indicative of vascular leak occur in dengue patients and are correlated with elevated chymase. The maximum (**a**) venous and (**b**) capillary hematocrit values that were recorded any time over a patient’s hospitalization and association with the study were higher in DHF patients compared to DF patients. (**c**) Compared to DF patients, DHF patients also had lower minimum platelet counts and (**d**) lower minimum pulse pressure recorded. (**e**) Elevated chymase is significantly associated with severely narrowed pulse pressure (< 20 mmHg). For (**a**–**e**) Student’s unpaired T test was used to compare DF versus DHF groups. (**f**) Serum chymase levels were significantly higher for patients with certain categories of warning signs (displayed at any time during involvement with the study) compared to patients in the DF no-WS group, as determined by 1-way ANOVA with Holm-Sidak’s post-test. For abdominal WS n = 24; vomiting n = 8; fluid accumulation n = 22; mucosal bleeding n = 3; lethargy n = 9; liver enlargement n = 22; hematologic WS n = 22; and severe leak n = 8. For all panels, error bars represent the SEM and * indicates *p* < 0.05; **p < 0.01; ***p < 0.001 and ****p < 0.0001.

Clinical signs and laboratory measures of disease, which were defined a priori, were obtained for hospitalized patients throughout their stays. Certain clinical parameters were also associated with DHF diagnosis, for example, DHF patients displayed higher maximum-recorded venous and capillary hematocrit values compared to DF patients (Fig. 3a,b). DHF patients also experienced significantly lower minimum platelet counts over the course of hospitalization (Fig. 3c). These hematological perturbations were expected since thrombocytopenia and plasma leakage are components of the DHF case definition. DENV patients also may experience narrowing of pulse pressure, which is strongly associated with hypovolemia in DHF Grades 3 and 4, or DSS^41^. We noted that the minimum pulse pressure throughout the patient’s involvement with the study was lower in DHF patients compared to DF patients (Fig. 3d). To evaluate whether narrowing of pulse pressure was also associated with elevated chymase levels, we compared the serum chymase concentrations between patients with narrow pulse pressure (below 20 mmHg) and those with pulse pressure > 20 mmHg. Indeed, serum chymase concentrations were significantly higher in patients with narrow pulse pressure (Fig. 3e).

To evaluate if any specific DENV warning signs were associated with elevated chymase, chymase levels were analyzed in patients who presented with one or more dengue warning sign. Interestingly, chymase levels were significantly higher in patients who presented with abdominal pain, fluid accumulation, lethargy, hematocrit increase concurrent with sudden platelet drop (hematologic WS) and/or severe leak, compared to the patients who did not present any warning signs (Fig. 3f). Chymase was not significantly elevated in DFWS patients displaying the warning signs of vomiting, mucosal bleeding, or liver enlargement compared to DF no-WS patients (Fig. 3f). This suggests a link between elevated chymase and DENV disease processes that involve pathologies of the vascular system and fluid accumulation.

### Higher chymase levels are associated with pleural effusion and gall bladder wall thickening in hospitalized dengue patients

**Figure 4:**
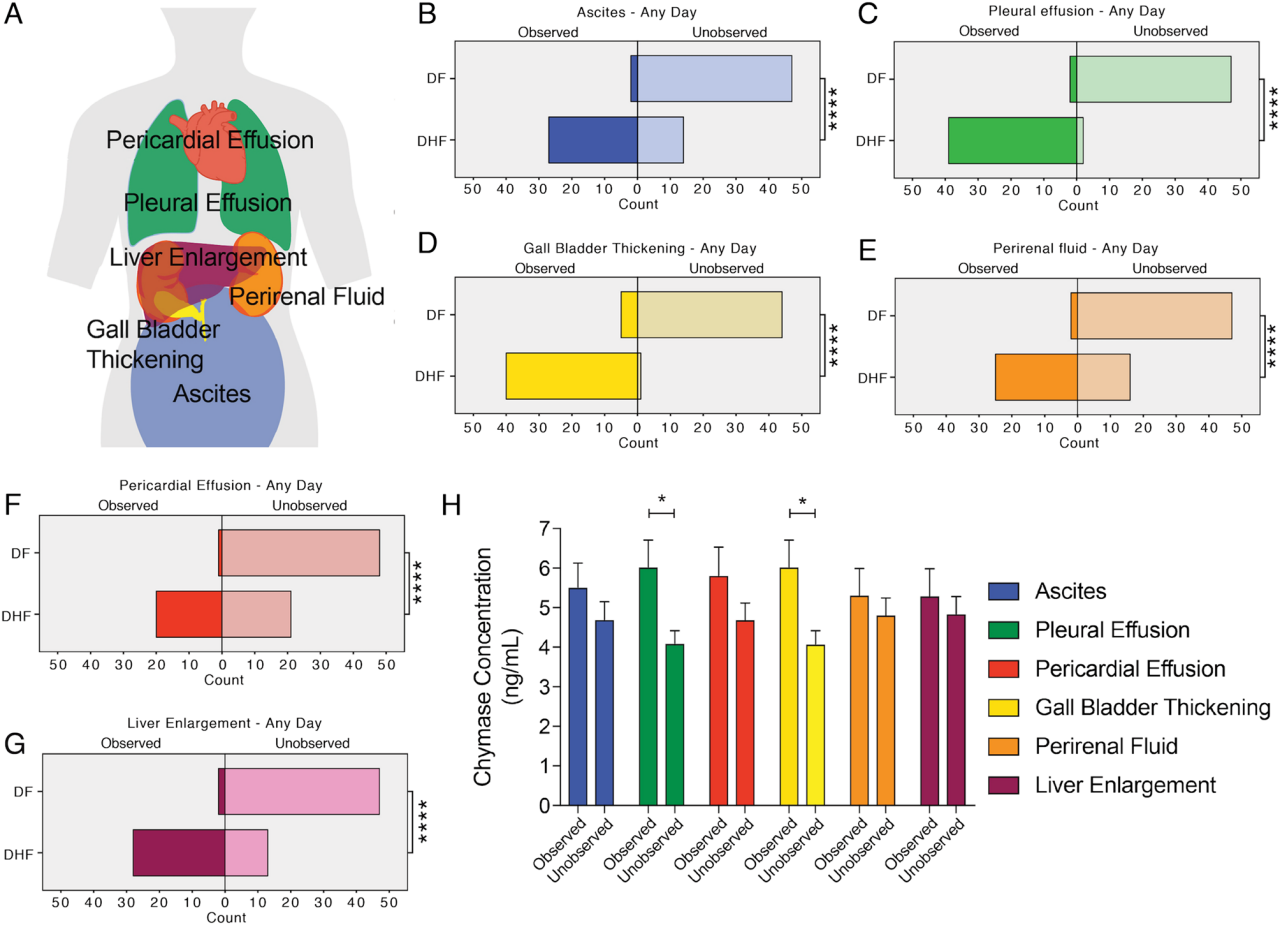
Elevated chymase correlates with observation of pleural effusion and gall bladder thickening in hospitalized dengue patients. (**a**) Patients in the study were given ultrasound exams daily, and observation of various indicators of DENV disease and/or fluid accumulation were recorded. Diagram was drawn using Adobe Illustrator CC software version 20.1.0. Proportions of patients where (**b**) ascites, (**c**) pleural effusion, (**d**) gall bladder thickening, (**e**) perirenal fluid accumulation, (**f**) pericardial effusion, or (**g**) liver enlargement were observed by ultrasound at any day of the study are presented for patients meeting the WHO-1997 diagnosis criteria of DF versus DHF. For (**b**–**g**) DHF patients displayed significantly increased incidence of observed abnormalities by ultrasound, as determined by Fisher’s exact test. (**** indicates *p* < 0.0001). (**h**) Serum chymase levels for patients where specific abnormal ultrasound results were observed versus unobserved were compared by 2-way ANOVA with Holm-Sidak’s post-test. * indicates *p* < 0.05. The observed power was calculated post-hoc to be 0.741. Error bars represent the SEM.

Plasma leakage during DHF can result in a life-threatening shock syndrome and is a consistent feature of severe dengue disease. Clinical and laboratory assessments indicated an association between fluid accumulation and chymase (Fig. 3); therefore, we aimed to confirm this using an independent technique to verify fluid accumulation. Some studies suggested that plasma leakage could be detected in DHF patients by ultrasound of the chest and abdominal cavity^42,43^. Thickening of the gall bladder wall and pericardial effusion have also been associated with plasma leak^20,44,45^. Therefore, we sought to verify if these were also observed in our pediatric cohort and/or correlated with chymase. By ultrasound, the extent of ascites, pleural effusion, gall bladder wall thickening, perirenal fluid accumulation, pericardial effusion and liver enlargement were assessed (Fig. 4a–g). For our analysis, we recorded whether these signs were observed by ultrasound at any time during the patient’s hospitalization. Indeed, all of these abnormal ultrasound observations were significantly associated with a DHF diagnosis (Fig. 4b–g). Interestingly, we observed that chymase levels were higher in patients where pleural effusion and/or gall bladder wall thickening were present, compared to patients where those signs were unobserved (Fig. 4h). This, in combination with the association of elevated chymase with hypovolemia (Fig. 3e), establishes chymase to be an important early indicator of the clinical fluid accumulation that is characteristic of DHF/DSS and Severe dengue.

## Discussion

Chymase, which is a vasoactive serine protease produced specifically by MCs, was found in this study to be significantly elevated in patients that experienced warning signs and laboratory parameters associated with vascular leakage. Prior human studies have indicated that chymase is an efficient biomarker for DHF prognosis since the protein levels are increased in the acute-phase serum samples from both adult and pediatric DENV cases that subsequently are diagnosed with DHF^33,35^. In contrast, virus-derived products such as NS1 or viremia have not consistently been shown to be predictive of severe disease^46,47^. In support of a mechanistic role for MC-derived products in DENV-induced vascular leakage, drugs that specifically inhibit MC activation are able to experimentally reduce vascular leakage in animal models^35,48^. In this prospective study, we aimed to provide more information regarding the association of chymase with severe dengue disease. We explored whether chymase levels correlate with measures of vascular leakage and clinical warning signs of disease severity. We also provide an understanding of chymase concentrations in the sera of hospitalized pediatric patients, longitudinally, and compare the ability of chymase to prognosticate both DHF and Severe dengue, according to the 1997 and 2009 WHO classification schemes. Our analysis showed that chymase was significantly associated with DHF and Severe dengue following both the WHO 1997 and 2009 guidelines for dengue classification.

As expected based on multiple studies comparing the two classification schemes^49-51^, reclassification of DHF patients in our cohort to the 2009 classification scheme resulted in two groups of patients: patients with Severe dengue and a subset of the DFWS patients, consistent with the view that the 2009 criteria more stringently define severity. Interestingly, while chymase was significantly elevated in the serum samples of patients at the time of presentation and predictive of both DHF and Severe dengue, the levels of chymase were similar for DHF/Severe patients and DHF/DFWS patients at early time points. These data support the potential utility of chymase as a prognostic biomarker of DHF/Severe dengue disease according to multiple classification schemes for disease severity. By the later time point of 5 days post-fever onset, DHF/Severe patients showed significantly higher levels of serum chymase than DHF/DFWS patients. This time point corresponds to an approximate time in the course of disease when fever begins to resolve, warning signs are more likely become apparent and severe disease may begin to present. The persistence of chymase in patients with Severe dengue compared to other groups may indicate a lack of resolution of disease processes promoting elevated chymase in the most severe patients. Furthermore, it raises the potential that chymase levels can not only be used to prognosticate DHF/Severe disease, but also to monitor for the effectiveness of therapeutic interventions.

This study revealed that signs and laboratory measures of vascular leakage, fluid accumulation and hypovolemia were strongly linked to elevated chymase. DHF patients displayed higher maximum venous and capillary hematocrit values compared to DF patients, as well as reduced platelet counts, as expected according to the case definition of DHF. Narrowing of pulse pressure was also significantly associated with DHF and those patients with narrow pulse pressure had higher levels of chymase than patients outside of the harmful pulse pressure range. Since none of the children in the study had cardiac-related pre-existing conditions and because narrow pulse pressure is a measure of dengue hypovolemia^52^, it suggests that early elevated chymase levels are linked to the subsequent presentation of hypovolemia. While these observations might suggest that chymase could also be a therapeutic target for severe DENV, our previous mechanistic studies suggest that its contributions to vascular leakage are less important than another protease, tryptase, which is also released during MC degranulation^34^, emphasizing that a biomarker need not be the most consequential mediator for a mechanism of disease to serve as a reliable indicator of disease.

Aside from laboratory measures, the presence of warning signs that are particularly indicative of vascular leakage were also linked to elevated chymase, including fluid accumulation, severe leakage and the hematologic warning sign of rapid rise in hematocrit concurrent with decreased platelets. Patients displaying the warning signs of abdominal pain and lethargy also had significantly higher levels of chymase compared to DF no-WS patients. We used ultrasounds to monitor the patients since multiple studies have indicated that DENV patients display abnormal ultrasound findings, such as gall bladder wall thickening and pleural effusion^43,53,54^. The majority of DHF patients displayed at least one abnormal ultrasound finding during the patients’ involvement within the study and abnormal ultrasounds were strongly associated with a diagnosis of DHF in this cohort. Significantly higher levels of serum chymase were noted in patients experiencing pleural effusion and gall bladder wall thickening, compared to patients where those signs were unobserved. Thus, ultrasound results support the association of chymase with fluid accumulation. However, liver involvement was not strongly associated with chymase levels or MC activation. We noted that the subset of patients that met the diagnostic criteria for DF/DFWS had lower levels of chymase compared to DHF/DFWS and a high proportion of liver enlargement observed as a key 2009 criteria warning sign. Consistent with this, patients with the warning sign of liver enlargement did not have higher levels of chymase compared to DF no-WS patients. Furthermore, chymase levels were not significantly higher in patients where liver enlargement by ultrasound was observed compared to patients where this was unobserved. The liver is a target organ of DENV infection and liver failure can occur as a component of multi-organ failure associated with DSS^55^. Although there are MCs present in the liver^56^, chymase levels are very low in this organ^57^, potentially indicating that liver involvement of DENV is less influenced by MCs.

There are multiple strengths of using this cohort to inform our understanding of the utility of chymase as a biomarker for severe DENV disease. First, we confined our analysis to prospectively recruited pediatric confirmed DENV patients that were hospitalized, allowing serial collection of serum samples and ultrasound readings and identification of warning signs that presented throughout the course of disease. However, we did not recruit patients for comparison with other febrile illnesses, which should also be compared to DENV patients in future studies. Importantly, chymase may be elevated in other infections that cause MC activation so we think chymase should be used for DENV prognosis when disease is highly suspected and confirmed by alternate laboratory tests. We also noted that chymase levels were higher on average in this patient cohort compared to a previous study we conducted in the same region of Sri Lanka^33^. We think that this is likely due to the fact that all of the patients studied here were hospitalized, likely leading to the recruitment of patients with more moderate/severe disease. Consistent with this notion, the levels of chymase in DF patients in this study were similar to the average chymase levels in DF patients with signs of bleeding that were previously assessed in a prior cohort and the baseline levels of healthy donors were also similar to those observed in prior studies^33^. We also previously reported that pediatric DENV patients had trends towards higher chymase levels during DHF compared to adults, although chymase was still prognostic for DHF in adults^33^. One limitation of this study was that for some clinical manifestations, particularly mucosal bleeding, the numbers of patients experiencing the sign were low. Therefore, further studies with a larger cohort are needed to verify the lack of association of mucosal bleeding with elevated chymase.

## Supplementary information

Supplementary file1.
