# Supplementary material for: Serum chymase levels correlate with severe dengue warning signs and clinical fluid accumulation in hospitalized pediatric patients
Source: Sci Rep. 2020 Jul 16;10:11856. doi: 10.1038/s41598-020-68844-z (PMC7367272; doi:10.1038/s41598-020-68844-z)
Supplement: Supplementary file 1 — Supplementary file1. [file 41598_2020_68844_MOESM1_ESM.pdf]

Serum chymase levels correlate with severe dengue warning signs and clinical fluid accumulation in hospitalized pediatric patients

Abhay P. S. Rathore[1,2#], Manouri Senanayake[3,4], Arjuna Athapathu[3], Sunethra Gunasena[5], Irantha Karunaratna[4], Wei Yee Leong[6], Ting Lim[1], Chinmay Kumar Mantri[1], Annelies Wilder-Smith[6,7,8], Ashley L. St. John[1,2,9,10]*

[1]Programme in Emerging Infectious Diseases, Duke-National University of Singapore, Singapore
[2]Department of Pathology, Duke University Medical Center, Durham, North Carolina, USA
[3]Department of Paediatrics, Faculty of Medicine, University of Colombo, Colombo, Sri Lanka
[4]Lady Ridgeway Children's Hospital, Colombo, Sri Lanka
[5]Department of Virology, Medical Research Institute (MRI), Colombo, Sri Lanka
[6]Lee Kong Chian School of Medicine, Nanyang Technological University, Singapore, Singapore
[7]London School of Hygiene and Tropical Medicine, Department of Disease Control, London, UK
[8]Heidelberg Institute of Global Health, University of Heidelberg, Heidelberg, Germany
[9]Department of Microbiology and Immunology, Yong Loo Lin School of Medicine, National University of Singapore, Singapore
[10]SingHealth Duke-NUS Global Health Institute, Singapore

[#]Current address

Keywords: Severe dengue, biomarker, chymase, plasma leakage, mast cells
Running title: Chymase correlation with severe dengue

*Correspondence may be addressed to:
Ashley St. John, Ph.D.
Program in Emerging Infectious Diseases
Duke-National University of Singapore Medical School
8 College Rd., Level 9
Singapore
Tel: +65 9771-7231
Email: ashley.st.john@duke-nus.edu.sg

Supplementary Figures:

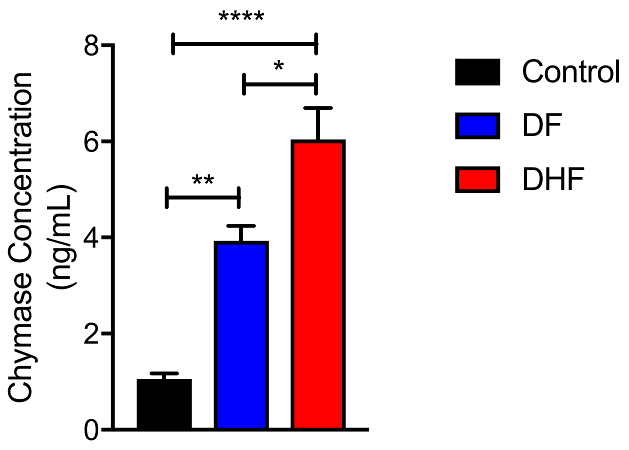

Figure S1: Comparison of serum chymase levels in dengue patients those of healthy controls.

The graph shows the serum chymase concentrations of healthy controls (n=10), DF, and DHF patients (defined according to the WHO 1997 criteria) at the day of enrolment in the study (Blood Sample 1). Statistical analysis was performed by 1-way ANOVA with Tukey's post-test. **** indicates $p<0.0001$, ** indicates $p<0.001$ and * indicates $p<0.01$. Error bars represent the SEM.

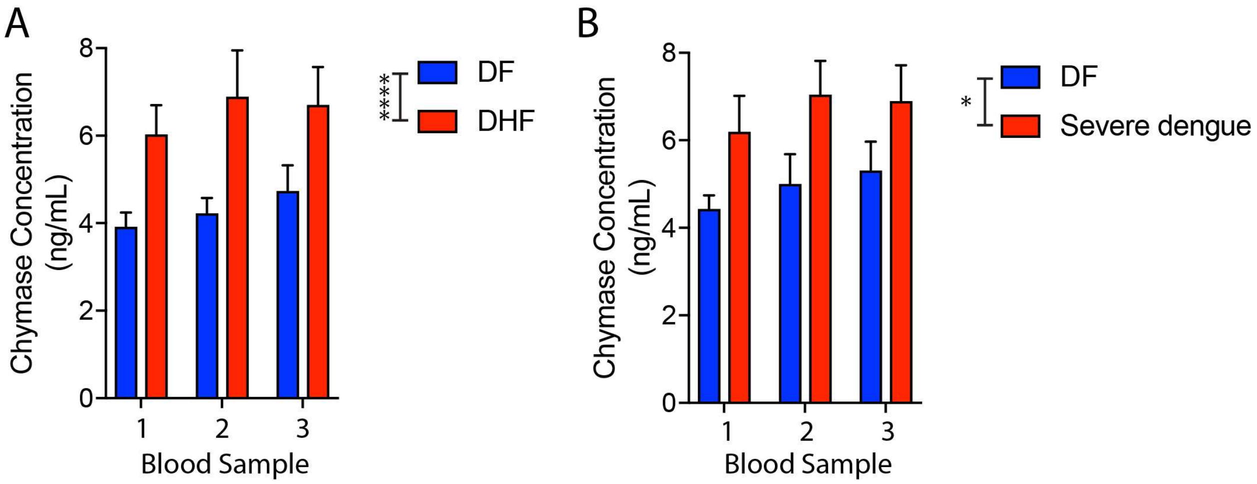

Figure S2: Serum chymase levels are prognostic of DHF and Severe dengue.

Comparisons of serum chymase concentrations between (**a**) DF and DHF patients (defined according to the WHO 1997 criteria), and (**b**) DF versus Severe dengue (defined according to the WHO 2009 criteria). Data are represented based on grouping relative to the day of enrolment (Blood Sample 1) and the subsequent sample collection days are shown as Blood Samples 2 and 3. Statistics were determined by 2-way ANOVA with Sidak's multiple comparison test. **** indicates $p<0.0001$, * indicates $p<0.01$. Error bars represent the SEM.

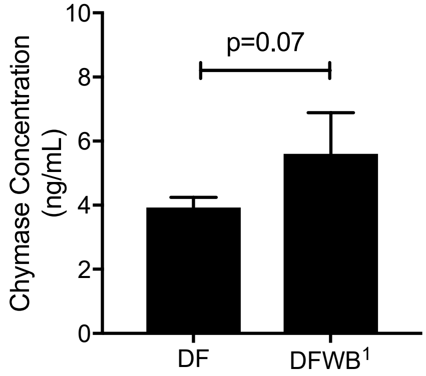

Figure S3: Chymase levels are similar in hospitalized DF patients to DF patients with bleeding (DFWB) in a prior study(1).

Levels of chymase in the serum of DF patients in this study (N=45) were compared to levels of chymase in patients designated as having DF with signs of bleeding (DFWB) from a prior study**(1)** in Sri Lanka (N=10). Data were compared using Student's unpaired T-test and error bars represent the SEM.

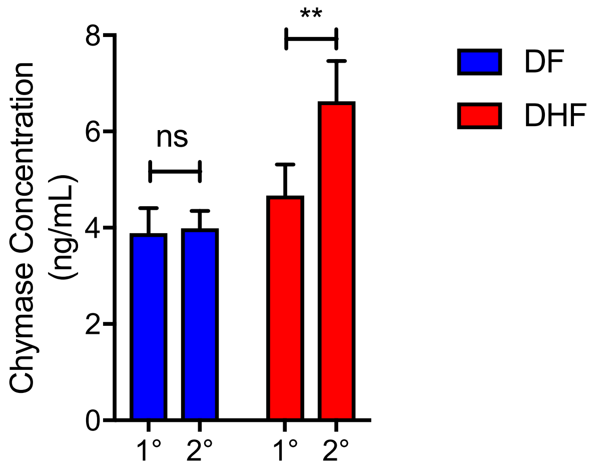

Figure S4: Elevated serum chymase in secondary compared to primary DHF patients.

Serum chymase levels were compared in primary (1°) and secondary (2°) DF and DHF patients at the time of enrollment in the study by 2-way ANOVA with Sidak's multiple comparison test, **p=0.0097. Error bars represent the SEM.

References

1. Tissera H, Rathore APS, Leong WY, Pike BL, Warkentien TE, Farouk FS, Syenina A, Ooi EE, Gubler DJ, Wilder-Smith A, St John AL. 2017. Chymase is a Predictive Biomarker of Dengue Hemorrhagic Fever in Pediatric and Adult Patients. J Infect Dis doi:10.1093/infdis/jix447.
